# Enhancing group lifestyle intervention for depression with ecological momentary assessment: a pilot randomized controlled trial

**DOI:** 10.1038/s41598-025-21688-x

**Published:** 2025-10-29

**Authors:** Vincent Wing-Hei Wong, Nga-Kwan Shi, Lavender Chui-Yiu Lee, Stephanie Wing-Yan Chan, Chee H. Ng, Jerome Sarris, Fiona Yan-Yee Ho

**Affiliations:** 1 Department of Psychology, The Chinese University of Hong Kong, Hong Kong, Hong Kong SAR; 2 Department of Psychiatry, The Melbourne Clinic and St Vincent’s Hospital, University of Melbourne, Richmond, VIC Australia; 3 NICM Health Research Institute, Western Sydney University, Westmead, NSW Australia; 4 The Florey Institute of Neuroscience and Mental Health & The Department of Psychiatry, The University of Melbourne, Melbourne, Australia

**Keywords:** Lifestyle, Depression, Group intervention, Ecological momentary assessment, Randomized controlled trial, Psychology, Depression

## Abstract

To investigate the impact of smartphone-delivered ecological momentary assessment (EMA) as a self-monitoring tool to complement a 6-week group face-to-face delivered multicomponent lifestyle medicine (LM) intervention for improving depressive symptoms. 56 Chinese Hong Kong adults with at least a moderate level of depressive symptoms were randomized to the EMA-supported intervention (LM/S; *n* = 18), pure intervention (PLM; *n* = 20), or care-as-usual (CAU; *n* = 18) groups. Data were collected at baseline, immediate post-intervention, and 3-month follow-up. LM/S only showed significantly greater vigorous physical activity than PLM at Week 19. At Week 7, PLM demonstrated a marginal reduction in depressive symptoms and significant improvements in insomnia symptoms, physical health-related quality of life (QoL), overall lifestyle, nutrition, and stress management compared to CAU, while LM/S improved only environmental health-related QoL. At Week 19, both intervention groups showed large improvements in depressive, anxiety, and insomnia symptoms, environmental health-related QoL, overall lifestyle, and stress management compared to CAU. Additional gains were observed for LM/S in nutrition, spiritual growth, and vigorous activity, and for PLM in physical and psychological health-related QoL, and interpersonal relationships. No significant differences in study attrition and intervention attendance were found between groups. Despite a low EMA compliance of 27.1%, the LM/S exhibited a higher, though not significantly different, full intervention adherence rate (66.67%) compared to the PLM (38.89%). A group-based, multicomponent LM intervention could potentially improve depressive symptoms, and smartphone-delivered EMA might enhance full intervention adherence despite modest compliance. A future adequately powered trial is warranted.

**Supplementary Information:**

The online version contains supplementary material available at 10.1038/s41598-025-21688-x.

## Introduction

Major depressive disorder (MDD) is characterized by depressed mood and loss of interest in activities, accompanied by various cognitive, behavioral, and physiological symptoms^1^. Globally, MDD is a significant public health concern affecting an estimated 280 million individuals^2,3^. Previous research has shown that depression is associated with reduced quality of life (QoL), impaired psychosocial functioning, and increased mortality risk from suicide^4–6^. According to the Global Burden of Diseases, Injuries, and Risk Factors Study 2019, MDD was found to be the leading cause of disability-adjusted life years (DALYs) among all mental disorders, accounting for 37.3% of the total burden^7^. Economically, MDD is associated with higher direct and indirect costs, such as inpatient treatment, emergency services, and loss of productivity across all age groups when compared to non-depressed individuals^8^. Taken together, the consequences of MDD are substantial, highlighting the need for effective prevention and treatment strategies to address this condition.

In recent years, a growing body of research has demonstrated that unhealthy lifestyle choices, such as physical inactivity and poor nutrition, are involved in the onset and development of MDD through several key biological pathways and processes, such as the stress response system and gut-brain axis^9,10^. As a result, there has been increasing interest in utilizing multicomponent lifestyle medicine (LM) interventions to prevent and manage MDD. Earlier investigations have supported the clinical utility of multicomponent LM interventions for improving depressive symptoms^9^. For example, initial evidence from a meta-analysis of randomized controlled trials (RCTs) involving 8,479 participants revealed that multicomponent LM interventions were efficacious for improving depressive symptoms relative to inactive controls at immediate post-intervention (*d*= 0.20–0.22)^11^. The authors suggested that the modest effect sizes might be attributable to the floor effect, given that the majority of the included RCTs targeted participants with mild depressive symptoms that were expected to have limited room for improvement. This position is reinforced by their subgroup analysis, which revealed a moderate effect size among those with MDD (*d*= 0.45). Consistent with Wong et al.^11^, recent RCTs have shown that multicomponent LM interventions for depression can be effectively delivered through diverse modes of delivery, such as in a group format^12^, via a smartphone application^10^, a website^13^, and a booklet series^14^, with moderate to large effect sizes (*d* = 0.66–0.94). With an expanding body of evidence, the LM approach has been recognized as an important treatment option for depression and has been incorporated into recent clinical guidelines for MDD^15–17^.

While multicomponent lifestyle modification interventions are shown to be efficacious for improving depressive symptoms, adherence to the intervention is often suboptimal. A meta-analysis of RCTs revealed that only 53% of the participants with depression fully adhered to the multicomponent LM interventions, which is considerably lower than the adherence rates observed for conventional treatments, such as face-to-face delivered cognitive behavioral therapy (84.7%)^18,19^. In line with Castro et al.^18^, our earlier pilot RCT, which examined the efficacy of a group-based multicomponent LM intervention (including diet, exercise, stress management, sleep management, and lifestyle psychoeducation) for individuals with at least moderate depressive symptoms, revealed that over 80% of participants did not fully adhere to the intervention^12^. Supporting service users with depression in adhering to the lifestyle intervention is of clinical importance, given that the LM approach posits that improvements in lifestyle-related diseases necessitate the active participation of service users in the modification process, and the therapeutic outcomes are contingent upon the extent of these lifestyle changes^20^. However, it could be particularly challenging for service users with depression, who commonly suffer from a lack of motivation, to actively implement lifestyle interventions and make sustained modifications in their lifestyle choices. In this context, health professionals play a crucial role in fostering self-efficacy, offering guidance, and ensuring service users remain engaged in the lifestyle modification process^20^.

While therapist guidance and dialogue support have been shown to enhance intervention adherence^18,21^, these approaches may be suboptimal in real-world settings due to the limited human resources and the high demand for mental health services^22^. An alternative approach to improve intervention adherence may be self-monitoring of lifestyle behaviors and mood, which involves systematically observing and recording the intended lifestyle behaviors (e.g., activity level, dietary intake, stress level, and sleep quality) and emotions (e.g., afraid, nervous, and upset) in one’s natural environment^23^. Previous studies have explored the potential underlying mechanism of self-monitoring as an adherence tool^24–26^. It is hypothesized that continuous self-monitoring could improve momentary self-awareness of one’s intended behaviors and emotions, thereby empowering individuals to take a more active role in and gain a deeper understanding of their personal health. This, in turn, may foster cognitive and behavioral changes that promote intervention adherence and, consequently, optimize intervention efficacy. A potential self-monitoring tool is a smartphone-delivered ecological momentary assessment (EMA)^26^. EMA entails repeatedly sampling the current behaviors and states of individuals in real time, making the responses gathered more ecologically valid with less recall bias. Given the ubiquitous nature of smartphones and technological advancements, recent research has utilized smartphones to collect EMA responses via various sampling protocols, which included interval-contingent, signal-contingent, and event-contingent designs^26^. Besides, the smartphone notification feature can also serve as a reliable way to remind participants to provide EMA responses.

Building on our previous work on group-based multicomponent LM intervention for depressive symptoms^12^, this pilot RCT was the first to investigate the impact of smartphone-delivered EMA as a self-monitoring tool to complement a multicomponent LM group intervention for improving depressive symptoms. This study hypothesized that participants randomly assigned to the EMA-supported group multicomponent LM intervention (LM/S) would demonstrate a significantly greater improvement in depressive symptoms, anxiety symptoms, insomnia symptoms, QoL, health-promoting behaviors (HPBs), functional impairment, and physical activity level relative to both the pure multicomponent LM intervention (PLM) and the care-as-usual (CAU) control group at immediate post-intervention (Week 7) and 3-month post-intervention follow-up assessments (Week 19). Additionally, we hypothesized that LM/S participants would show a significantly lower study attrition rate but higher intervention attendance, credibility, and expectancy in relation to PLM and CAU. Besides, we hypothesized that the EMA compliance rate for the LM/S group would be comparable to existing trials targeting the promotion of health behaviors.

## Method

This pilot three-arm, parallel-group RCT was conducted at the Department of Psychology, The Chinese University of Hong Kong, Hong Kong. Ethical approval was sought from the Joint Chinese University of Hong Kong – New Territories East Cluster Clinical Research Ethics Committee (The Joint CUHK-NTEC CREC) (Reference No. 2021.369), and the trial protocol was pre-registered on ClinicalTrials.gov (Reference No. NCT04875663; Registration Date: 06/05/2021). This study followed the Consolidated Standards for Reporting Trials (CONSORT) statement: extension to randomized pilot and feasibility trials for reporting^27^.

### Study population and procedure

A total of 56 participants were recruited through printed posters at the CUHK, the CUHK mass mail system, word of mouth, and advertisements on social media platforms, including Facebook and Instagram. Eligible participants were Hong Kong residents who met the following criteria: (1) aged 18 years or older; (2) had at least moderate depressive symptomatology, as indicated by a Patient Health Questionnaire-9 (PHQ-9) total score ≥ 10^28^; (3) possessed an internet-enabled mobile device, compatible with either iOS or Android operating systems; (4) demonstrated fluency in Cantonese; (5) were able to comprehend Chinese; and (6) agreed to provide informed consent and adhere to the trial protocol. Participants were excluded if they: (1) presented a current serious suicidal risk (non-fleeting intent or plan) as assessed by a PHQ-9 Item 9 score > 2 (information for professional mental health service referrals was provided); (2) had any medical or neurocognitive disorder(s) that, based on the research team’s clinical experience, made participation unsuitable or might interfere with adherence to the lifestyle modifications (e.g., exercise or dietary changes were not recommended by physicians); (3) were currently involved in professionally supervised lifestyle changes; (4) had unstable medication in the past 3 months or were receiving psychotherapy for depression; (5) were pregnant; (6) were hospitalized; and (7) enrolled in any other trial(s).

Prospective participants were asked to complete a set of online screening questionnaires on an online survey platform, which included (1) PHQ-9 assessing the level of depressive symptoms and suicidal ideation; (2) a self-report checklist for eligibility criteria; and (3) a non-validated demographic questionnaire. Eligible participants were subsequently invited to join the study by a research assistant through an approximately 20-minute phone call. During the call, eligible participants were informed about the study objective, procedures, restrictions, data handling, privacy measures, potential risks, and the voluntary nature of participation. Interested participants were guided to download an in-house smartphone application (*Longitudinax Pro*) to provide electronic informed consent and for outcome data collection. Upon granting consent and completing the baseline assessment, participants were randomly assigned to the LM/S (*n* = 18), PLM (*n* = 20), or CAU (*n* = 18) groups with a ratio of 1:1:1 by an independent statistician using a computer-generated list of numbers. Due to the nature of the trial design, it was not feasible to blind the intervention allocation from participants and therapists. To ensure unbiased data regarding participants’ attitudes and behaviors, incomplete disclosure was employed by withholding information about the EMA component in this study. Specifically, during the informed consent process, all participants were told that this RCT consisted of only 2 arms, i.e., a group-based LM intervention arm and a CAU arm. The EMA component was withheld from participants in the PLM and CAU groups throughout the trial. In contrast, those in the LM/S group believed that all participants receiving the group multicomponent LM intervention had access to the smartphone-delivered EMA application. After completing the 3-month follow-up assessment (Week 19), a debriefing session was held to reveal the use of EMA in the study and explain the rationale behind withholding this information.

After randomization, the research assistant contacted participants in the LM/S and PLM groups via text messages to discuss group intervention arrangements, while participants in the CAU group were informed of the dates for the immediate post-intervention (Week 7) and 3-month follow-up assessments (Week 19). Throughout the trial period, participants were able to contact the research assistant solely for non-therapeutic support. This included addressing technical issues related to the *Longitudinax Pro*, clarifying assessment dates, and notifying the research assistant about their attendance of intervention sessions. To acknowledge participants’ time and involvement, those who completed all study and assessment procedures received compensation of HK$200 (equivalent to US$26).

### The LM intervention

Participants allocated to the LM/S and PLM groups received a 6-week group-based LM intervention. Each 2-hour session (i.e., 12 hours in total) was conducted in a group of 8–10 participants to promote group discussion and interaction. The manualized intervention was administered by a multidisciplinary team under the regular supervision of a doctoral-level clinical psychologist (F.Y.H.) and board-certified lifestyle medicine professionals (V.W.W. and F.Y.H.). The team also included a clinical psychology trainee, a registered dietitian, and a physical trainer. To ensure the intervention content was adequately delivered, F.Y.H. observed each session with subsequent feedback provided to the therapists.

The group-based LM intervention was informed by the five stages of change outlined in the Transtheoretical Model (TTM)^29^ and designed by the research team drawing upon clinical guidelines and up-to-date research findings^15–17^. The intervention protocol included 4 core lifestyle modules (i.e., “Core Module 1: Diet and Nutrition”, “Core Module 2: Physical Activity”, “Core Module 3: Sleep Management”, and “Core Module 4: Stress Management”) aiming to enhance participants’ mental health and well-being. In addition, 2 cross-cutting modules (i.e., “Cross-Cutting Module 1: Motivational Interviewing” and “Cross-Cutting Module 2: Goal Setting”) were designed to engage participants in ongoing lifestyle modifications and promote intervention adherence. The intervention content was verified by qualified clinical psychologists, psychiatrists, dietitians, and physical instructors. In “Core Module 1: Diet and Nutrition”, the dietitian provided evidence-based nutrition information for improving and maintaining mental health. This module covered (1) the connection between diet and mental health, (2) recommendations for a healthy and balanced diet, (3) interpreting nutrition facts labels, (4) integrating a healthy diet into daily life, and (5) action steps for adopting a healthy diet. In “Core Module 2: Physical Activity”, the physical instructor introduced and demonstrated various types of gentle and adaptable exercises during the session (i.e., low-intensity home-based and outdoor activities) and provided simple methods to assist participants in incorporating exercises into their daily routines. Besides, the physical instructor provided physical activity recommendations and discussed the physical and psychological benefits of regular exercise. Concerning “Core Module 3: Sleep Management”, basic information about sleep, the vicious cycle of insomnia, and the relationship between circadian and social rhythms in connection with sleep and mental health were introduced. Practical skills, such as sleep hygiene, regular sleep-wake times, wind-down routine, and daytime functioning improvement, were also included. Regarding “Core Module 4: Stress Management”), the clinical psychology trainee delivered psychoeducation on stress, worry management, gratitude cultivation, and relaxation techniques, including diaphragmatic breathing and progressive muscle relaxation. Furthermore, the 2 cross-cutting modules were implemented to boost motivation, facilitate long-term adherence, and foster enjoyment of the skills learned throughout the program. The content encompassed areas such as examining current goals, the motivation matrix, SMART goal setting, and goal evaluation and maintenance.

To facilitate effective lifestyle modifications and ensure a smooth transition, the intervention content was delivered in accordance with the change process outlined in the TTM^29^. A detailed intervention structure is depicted in Supplementary Table 1. In each session, the therapists reviewed the participants’ weekly logs and homework from the previous session (except for the first session), delivered core intervention content, assigned homework for the next session, summarized and highlighted the key points of the session, asked participants to provide feedback, and discussed potential barriers to lifestyle modifications. An intervention handout, including session content and homework, was distributed for review in each session.

In addition to the group-based LM intervention, participants in the LM/S group had access to an in-house smartphone-delivered EMA application (the app) for the 6-week intervention period (i.e., 42 days). The app utilized interval-contingent assessments, prompting participants to complete surveys at 4 fixed time points each day (i.e., 12 pm, 3 pm, 6 pm, 9 pm), with automatic reminders sent at 5-minute intervals (up to four reminders per time point) to encourage timely responses. The EMA app captured real-time data across 5 health-related domains, including diet, physical activity, sleep management, stress management, and mood state. For diet, participants reported whether they had eaten since the last check-in. If yes, they specified the type of intake (i.e., food, drinks, snacks, or supplements), uploaded a photo documenting their planned and unplanned intake, and rated the perceived meal quality on a 11-point Likert scale (0–10). For physical activity, participants recorded the number of minutes engaged in light, moderate, and vigorous exercise at each time point. Sleep assessments included a daily rating of overall sleep quality on an 11-point Likert scale (0–10) at 12 pm and repeated ratings of current sleepiness at all time points. Stress management was assessed by tracking relaxation practices (including type and duration) at each time point, with an additional daily rating of overall stress intensity at 9 pm on an 11-point Likert scale (0–10). Finally, mood was measured at all time points using the Chinese version of the International Positive and Negative Affect Schedule Short Form (I-PANAS-SF), which includes 10 items rated on a 5-point Likert scale ranging from 1 (never) to 5 (always)^30^, capturing both positive and negative affective states. In each weekly session, therapists spent the initial 15 min reviewing participants’ adherence to lifestyle recommendations and discussing potential facilitators and barriers to lifestyle modifications.

### The CAU

The CAU group did not receive the group-based LM intervention. Instead, they accessed care based on their needs and preferences, which included but were not limited to pharmacological interventions, psychological interventions, and complementary and alternative medicine. In addition, the research assistant reminded participants in the CAU to adhere to the study protocol. Upon completing follow-up assessments at Week 19, the CAU group was provided with a smartphone-delivered LM intervention^10^.

### Data collection

All participants completed a set of online questionnaires at baseline (Week 0), immediate post-intervention (Week 7), and 3-month post-intervention follow-up assessments (Week 19). The primary outcome was the mean difference in depressive symptoms from baseline to Week 7. Specifically, the main comparisons were made between the LM/S and PLM, LM/S and CAU, and PLM and CAU groups. The secondary outcomes included anxiety symptoms, perceived insomnia severity, QoL, HPBs, functional disability, level of physical activity, sociodemographic characteristics, intervention expectancy and credibility, study attrition (i.e., the rate of participant withdrawal throughout the entire research period), intervention attendance (i.e., the mean group sessions attended and the proportion of participants with full intervention attendance), and EMA compliance rate.

### Screening and outcome measures

The Chinese version of the PHQ-9 was used to measure the severity of depressive symptoms in the past 2 weeks^28,31^. The PHQ-9 is a 9-item self-report questionnaire rated on a 4-point Likert scale, with 4 possible response categories: not at all (0), several days (1), more than half the days (2), and nearly every day (3). The total score is the sum of the 9-item scores and can be divided into 5 categories: no symptoms (0–4), mild symptoms (5–9), moderate symptoms (10–14), moderately severe symptoms (15–19), and severe symptoms (20–27). A total score ≥ 10 was considered as a probable clinical case^32,33^. The PHQ-9 demonstrated good psychometric properties in the present study (Cronbach’s alpha = 0.87).

The Chinese version of the Generalized Anxiety Disorder-7 assessment (GAD-7) was employed to assess the severity of generalized anxiety symptoms in the past 2 weeks^34,35^. The GAD-7 is a 7-item self-report questionnaire rated on a 4-point Likert scale, with 4 possible response categories: not at all (0), several days (1), more than half the days (2), and nearly every day (3). The total score is the sum of the 7-item scores and can be divided into 4 categories: minimal symptoms (0–4), mild symptoms (5–9), moderate symptoms (10–14), and severe symptoms (15–21). The GAD-7 demonstrated good psychometric properties in the present study (Cronbach’s alpha = 0.92).

The Chinese version of the Insomnia Severity Index (ISI) was used to measure the severity of insomnia symptoms^36,37^. The ISI is a 7-item self-report questionnaire rated on a 5-point Likert scale, with 5 possible response categories for each question: none/very satisfied (0), mild/satisfied (1), moderate/moderately satisfied (2), severe/dissatisfied (3), and very severe/very dissatisfied (4). The total score is the sum of the 7-item scores and can be divided into 4 categories: no clinically significant symptoms (0–7), subthreshold symptoms (8–14), moderate symptoms (15–21), and severe symptoms (22–28). The ISI demonstrated good psychometric properties in the present study (Cronbach’s alpha = 0.88).

The Chinese version of the World Health Organization Quality of Life Instruments (WHOQOL-BREF) was utilized to assess the QoL in terms of physical health, psychological, social relationships, environment, overall QoL, and general health^38,39^. The WHOQOL-BREF consists of 26-item rated on a five-point ordinal scale ranging from 1 to 5. Following the scoring guidelines, the scores were transformed into a linear scale from 0 to 100. A higher score indicates better QoL. The WHOQOL-BREF demonstrated good psychometric properties in the present study (Cronbach’s alpha = 0.94).

HPBs were measured by the Chinese version of the Health-Promoting Lifestyle Profile (HPLP-II)^40,41^. HPLP-II is a 52-item self-report questionnaire assessing six domains of lifestyle-related behaviors, which included spiritual growth (9 items), interpersonal relations (9 items), nutrition (9 items), physical activity (8 items), health responsibility (9 items), and stress management (8 items). All items are rated on a 4-point Likert scale with 4 possible response categories: never (1), sometimes (2), frequently (3), and regularly (4). The total and domain scores are the mean of the respective item scores. The HPLP-II demonstrated good psychometric properties in the present study (Cronbach’s alpha = 0.71–0.96).

The Chinese version of the Sheehan Disability Scale (SDS) was used to measure the level of functional impairment^42,43^. The SDS is a 3-item self-report questionnaire that measures functional impairment in work/school, social life, and family life. Each item is measured on a 10-point Likert scale, of which 0 represents no disruption and 10 represents extreme disruption. The total score is the sum of the 3-item scores. The SDS demonstrated good psychometric properties in the present study (Cronbach’s alpha = 0.92).

Physical activity level was evaluated by the International Physical Activities Questionnaire – Chinese version (IPAQ-C)^44,45^. The IPAQ-C captures information on various types and intensity levels of physical activities, including walking, moderate-intensity, and vigorous-intensity activities, and time spent sitting in the past 7 days via 7 items. The total activity level was calculated by considering the time spent in each activity intensity and its corresponding metabolic equivalent (MET) energy expenditure, of which walking equals 3.3 METs, moderate activities equal 4 METs, and vigorous activities equal 8 METs^46^. The IPAQ-C demonstrated good psychometric properties in the present study (Cronbach’s alpha = 0.85).

The Chinese version of the Credibility-Expectancy Questionnaire (CEQ) was used to evaluate intervention expectancy and credibility^47^. The CEQ is a 6-item scale, with the sum of the first 3 items providing a measure of intervention credibility and the sum of the remaining 3 items examining intervention expectancy. Higher scores indicate greater credibility and success expectancy for the intervention. The CEQ demonstrated good psychometric properties in the present study (Cronbach’s alpha = 0.72–0.88).

A non-validated demographic survey was used to collect sociodemographic information, including age, sex, level of education, employment status, monthly income level, and marital status.

### Statistical analysis

The sample size for this study was determined in accordance with recommendations for pilot clinical trials, which suggest including at least 12 participants per group^48^. The R Statistical Software v4.3.0^49^ was used for all statistical analyses. A *p*-value of less than .05 (two-tailed) was used to determine statistical significance. Effect sizes were calculated using Cohen’s *d*, which is determined by dividing the estimated mean differences by the pooled standard deviation (*SD*)^50^. According to Cohen (2013),^51^ effect sizes of 0.2, 0.5, and 0.8 were considered small, medium, and large, respectively. Baseline characteristics were summarized as means and *SD*s for approximately normally distributed continuous variables and as median and interquartile range (IQR) for skewed variables. Categorical variables were reported as frequencies and percentages. Baseline differences across groups were examined using one-way ANOVA for normally distributed continuous variables and the Kruskal–Wallis test for non-normal variables. For categorical variables, chi-square tests (*x*^2^) was used when fewer than 20% of expected cell counts were < 5 and no cell had an expected count < 1. When these assumptions were violated, Fisher’s exact test was employed^52^. Normality was evaluated using Q-Q plots and the Shapiro–Wilk test.

Based on the intention-to-treat (ITT) approach, the group (LM/S, PLM, and CAU) by time (Week 0, Week 7, and Week 19) interactions for the primary (i.e., depressive symptoms) and secondary outcomes (i.e., anxiety symptoms, insomnia symptoms, QoL, HPBs, functional impairment, physical activity levels) were evaluated using linear mixed-effects models. Significant baseline characteristics were included as covariates in the models to account for potential confounding effects and ensure comparability across groups. Missing data were handled via maximum likelihood estimation under the assumption that data were missing at random^53,54^. To verify model assumptions, we assessed normality using Q-Q plots and the Shapiro–Wilk test, and homogeneity of variance with Levene’s test. Due to observed deviations from the normality assumption for residuals, we utilized a robust linear mixed-effects model (RLMM) implemented using the ‘robustlmm’ package in R, which is specifically designed to provide robust parameter estimation in the presence of outliers or deviations from model assumptions. Tukey’s Honestly Significance Difference (HSD) post-hoc pairwise comparisons were conducted using the estimates derived from the RLMM models with *p*-value adjusted for multiple testing. In addition, RLMMs were used to examine intervention credibility and expectancy from Week 0 to Week 7.

The study attrition rates of the 3 groups were summarized as percentages. The Fisher’s exact test was utilized to examine whether dropout rates differed significantly between the 3 groups. The mean intervention adherence rates between the 2 intervention groups were compared using a Mann-Whitney U test, while the proportion of participants with full intervention attendance between the 2 intervention groups was compared using Fisher’s exact test. Clinically significant improvement was defined as a minimum 5-point reduction of the PHQ-9 total score from Week 0 to Week 7^55^. In addition, we examined the proportion of participants who achieved a PHQ-9 total score of < 10 between the 3 groups at Week 7. The Fisher’s exact tests were used to analyze the between-group difference in the proportion of participants who attained clinically significant improvement and a PHQ-9 total score of < 10.

Additionally, we assessed the acceptability of using smartphone-delivered EMA as a self-monitoring tool for lifestyle behaviors and mood in terms of compliance rates and fatigue effects (i.e., response rates to EMA across the 6 intervention weeks)^26,56^. The compliance rates of EMA were summarised at the sample and weekly levels^56^. Participants who achieved the minimum compliance threshold of the EMA measure (i.e., completed ≥ 30% of the total EMA measure) were classified as ‘completers’, otherwise they were considered as ‘non-completers’^56–58^. Mann-Whitney U test and Fisher’s exact test was conducted to identify any significant difference in baseline measures between ‘completers’ and ‘non-completers’. In addition, a Friedman test was performed to examine the fatigue effects based on weekly EMA compliance rates.

## Results

### Participant characteristics

**Fig. 1:**
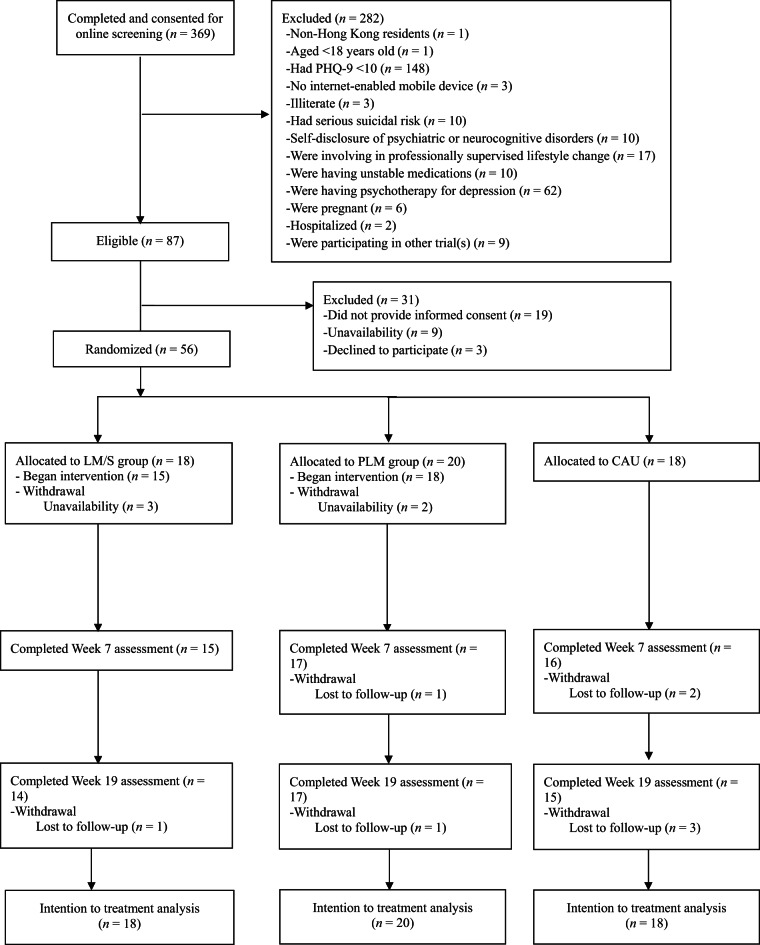
Study flow diagram.

A total of 369 individuals completed the online screening. Of these, 282 individuals were excluded for various reasons (see Fig. 1 for details). The remaining 87 eligible individuals were invited to participate in the study. However, 19 of them did not provide informed consent, 9 were unavailable, and 3 declined to participate without specifying a reason. In sum, 56 participants were randomly assigned to the LM/S (*n* = 18), PLM (*n* = 20), and CAU (*n* = 18), respectively. The mean age of the total sample was 39.36 years (*SD* = 10.84), and the majority were female (85.71%) and held a university education (58.92%). Statistically significant baseline differences between the 3 groups were observed in educational level (*p* <.05) and QoL related to physical health (*p* <.01). No other baseline clinical, lifestyle, or psychosocial characteristics showed significant differences between groups (*p*s > .05) (Table 1).

### Study attrition and intervention adherence

The Fisher’s exact test indicated no significant differences in study attrition rates between LM/S (*n* = 4, 22.22%), PLM (*n* = 3, 15%), and CAU (*n* = 3, 16.67%) groups (*p* =.91). Detailed reasons for attrition are present in Fig. 1. Regarding intervention adherence, LM/S participants completed an average of 5.2 out of 6 sessions (i.e., 86.67%, range = 2–6), while PLM participants completed an average of 5 out of 6 sessions (i.e., 83.33%, range = 3–6). The Mann-Whitney U test showed no significant difference in intervention attendance between the LM/S and PLM groups (*U* = 163, *p* =.28). With respect to the proportion of participants demonstrating full adherence to the intervention sessions, 10 LM/S (i.e., 66.67%) and 7 PLM (38.89%) participants had completed all the intervention sessions. The Fisher’s exact test indicated no significant differences in the proportion of participants demonstrating full adherence to the intervention sessions between LM/S and PLM (*p* =.33).

**Table 1:** Baseline clinical, lifestyle, and psychosocial characteristics.

Variable	LM/S (*n* = 18)	PLM (*n* = 20)	CAU (*n* = 18)	Total (*n* = 56)	*p*-value
Age, years [mean (*SD*)]	40.06 (9.69)	40.45 (11.74)	37.44 (11.24)	39.36 (10.8)	.22
Female, *n* (%)	15 (83.3)	19 (95)	14 (77.8)	48 (85.7)	.32
Marital status, *n* (%)					.72
Not married	12 (66.6)	14 (70)	10 (55.6)	36 (64.3)	
Married	6 (33.3)	5 (25)	6 (33.3)	17 (30.4)	
Divorced/widowed	0 (0)	1 (5)	2 (11.1)	3 (5.4)	
Educational level, *n* (%)					< .05*
Secondary or below	8 (44.4)	6 (30)	2 (11.1)	16 (28.6)	
Associate degree, diploma, or vocational training	5 (27.8)	0 (0)	2 (11.1)	7 (12.5)	
Bachelor’s degree	4 (22.2)	12 (60)	11 (61.1)	27 (48.2)	
Master’s degree or above	1 (5.6)	2 (10)	3 (16.7)	6 (10.7)	
Number of children, *n* (%)					.60
0	15 (83.3)	15 (75)	15 (83.3)	45 (80.4)	
1	2 (11.1)	2 (10)	3 (16.7)	7 (12.5)	
≥ 2	1 (5.6)	3 (15)	0 (0)	4 (7.1)	
Employment status, *n* (%)					.51
Full-time work/student	12 (66.7)	13 (65)	13 (72.2)	38 (67.9)	
Part-time work	2 (11.1)	3 (15)	1 (5.6)	6 (10.7)	
Unemployed	0 (0)	2 (10)	3 (16.7)	5 (8.9)	
Retired or carer	4 (22.2)	2 (10)	1 (5.6)	7 (12.5)	
Monthly income, *n* (%)					.85
≤HKD$ 5,000	5 (27.8)	5 (25)	4 (22.2)	14 (25)	
HKD$ 5,001–10,000	3 (16.7)	3 (15)	1 (5.6)	7 (12.5)	
HKD$ 10,001–20,000	3 (16.7)	5 (25)	4 (22.2)	12 (21.4)	
HKD$ 20,001–30,000	2 (11.1)	3 (15)	6 (33.3)	11 (19.6)	
HKD$ 30,001–50,000	3 (16.7)	2 (10)	3 (16.7)	8 (14.3)	
HKD$ 50,001–70,000	2 (11.1)	1 (5)	0 (0)	3 (5.4)	
HKD$ 70,001–90,000	0 (0)	1 (5)	0 (0)	0 (0)	
> HKD$ 90,000	0 (0)	0 (0)	0 (0)	1 (1.79)	
Current antidepressant medication, *n* (%)	1 (11.1)	1 (10)	1 (5.6)	5 (8.93)	.99
PHQ-9 [median (IQR)]	9.5 (9.25)	10.5 (5.5)	11.5 (4.75)	11 (4.2)	.53
GAD7 [mean (*SD*)]	14.94 (4.52)	11.65 (4.99)	10.89 (4.34)	11.38 (4.7)	.67
ISI [mean (*SD*)]	11.56 (4.94)	11.7 (4.43)	13.56 (5.39)	13.34 (4.89)	.40
SDS [median (IQR)]	8.5 (13.25)	10 (10.5)	13 (9.75)	10 (12.5)	.32
WHOQOL-brief					
Physical health [mean (*SD*)]	2.9 (0.46)	2.69 (0.39)	2.45 (0.4)	2.68 (0.45)	< .01**
Psychological health [mean (*SD*)]	2.73 (0.57)	2.55 (0.38)	2.62 (0.48)	2.63 (0.48)	.49
Social relationship [mean (*SD*)]	2.74 (0.82)	2.97 (0.52)	2.93 (0.8)	2.88 (0.71)	.44
Environmental health [mean (*SD*)]	3.13 (0.64)	2.88 (0.49)	2.97 (0.67)	2.99 (0.60)	.41
Quality of life [median (IQR)]	3 (1.5)	3 (0)	3 (1.75)	3 (1)	.87
General health [median (IQR)]	2 (1)	2 (0.25)	2 (1)	2 (1)	.26
HPLP-II					
Total score [mean (*SD*)]	106.11 (15.85)	103.2 (16.92)	104.28 (22.64)	104.48 (18.34)	.77
Health responsibility [median (IQR)]	17(4)	15.5 (5.5)	17 (4.75)	17 (6)	.52
Physical activity [median (IQR)]	16.5 (7)	13 (4)	15.5 (4.75)	14.5 (5.25)	.08
Nutrition [mean (*SD*)]	19.72 (3.44)	20.5 (5.44)	20.28 (5.28)	20.18 (4.76)	.73
Spiritual growth [mean (*SD*)]	18.44 (4.67)	17.1 (3.77)	16.33 (5.44)	17.29 (4.64)	.17
Interpersonal relationship [mean (*SD*)]	18.78 (2.67)	20.3 (4.46)	18.61 (5.07)	19.27 (4.2)	.91
Stress management	15.5 (3.75)	14 (5)	15 (4.5)	15 (4.25)	.71
IPAQ					
Total activity [median (IQR)]	1470 (3217.5)	933 (829.5)	1272 (840.5)	1222.5 (1110)	.20
Vigorous activity [median (IQR)]	720 (960)	0 (360)	400 (960)	400 (960)	.09
Moderate activity [median (IQR)]	160 (420)	0 (240)	160 (480)	120 (360)	.45
Walking [median (IQR)]	1386 (1798.5)	693 (478.5)	495 (330)	693 (990)	.18
CEQ					
Credibility [mean (*SD*)]	19.17 (3.9)	16.45 (4.42)	17 (4.87)	17.50 (4.49)	.15
Expectancy [mean (*SD*)]	17.06 (3.98)	13.05 (3.19)	15.13 (4.25)	15.01 (4.09)	.16

CAU, care-as-usual: CEQ, Credibility-Expectancy Questionnaire; LM/S, EMA-supported group multicomponent LM intervention: GAD-7, Generalized Anxiety Disorder-7 assessment; HPLP-II, Health-Promoting Lifestyle Profile; IPAQ, International Physical Activities Questionnaire – Chinese version; IQR, interquartile range; ISI, Insomnia Severity Index; PHQ-9, Patient Health Questionnaire; PLM, pure multicomponent LM intervention; SD, standard deviation; SDS, Sheehan Disability Scale; WHOQOL-brief, World Health Organization Quality of Life Instruments.

* *p* <.05.

** *p* <.01.

### Primary outcome

At Week 7, LM/S did not demonstrate significant improvement compared to CAU in depressive symptoms (adjusted mean difference = − 1.21, *p* =.80, *d* = 0.24), while the PLM showed a marginally significant reduction relative to CAU (adjusted mean difference = − 3.96, *p* =.05, *d* = 0.78). At Week 19, both LM/S (adjusted mean difference = − 5.80, *p* <.01, *d* = 1.14) and PLM (adjusted mean difference = − 4.02, *p* <.05, *d* = 0.79) demonstrated significantly greater reductions in depressive symptoms relative to CAU. No significant difference was observed between LM/S and PLM at Weeks 7 and 19 (Table 2).

### Secondary outcomes

At Weeks 7 and 19, no significant differences were observed between LM/S and PLM in all secondary outcomes, except that LM/S yielded significantly greater IPAQ-assessed vigorous physical activity level than PLM at Week 19 (adjusted mean difference = 745.03, *p* <.01, *d* = 0.71). At Week 7, relative to CAU, LM/S showed a significant improvement only in environmental health-related QoL (adjusted mean difference = 0.55, *p* <.05, *d* = 0.92), whereas PLM significantly improved insomnia symptoms (adjusted mean difference = −5.40, *p* <.01, *d* = 1.13), physical health-related QoL (adjusted mean difference = 0.32, *p* <.01, *d* = 0.76), overall lifestyle (adjusted mean difference = 16.77, *p* <.05, *d* = 0.90), nutrition (adjusted mean difference = 4.34, *p* <.05, *d* = 0.90), and stress management (adjusted mean difference = 3.07, *p* <.05, *d* = 0.89) compared to CAU.

At Week 19, both LM/S and PLM demonstrated significant improvements compared to CAU in anxiety symptoms (adjusted mean difference = −4.82 to −5.22, *ps* < 0.05, *d* = 1.09 to 1.10), insomnia symptoms (adjusted mean difference = −6.21 to −6.90, *ps* < 0.001, *d* = 1.30 to 1.44), environmental health-related QoL (adjusted mean difference = 0.50 to 0.74, *ps* < 0.05, *d* = 0.84 to 1.23), overall lifestyle (adjusted mean difference = 20.25 to 21.16, *ps* < 0.01, *d* = 1.09 to 1.14), and stress management (adjusted mean difference = 2.86 to 3.62, *ps* < 0.05, *d* = 0.83 to 1.05). Additionally, LM/S significantly improved nutrition (adjusted mean difference = 4.81, *p* <.05, *d* = 1.00), spiritual growth (adjusted mean difference = 4.44, *p* <.05, *d* = 0.95), and IPAQ-assessed vigorous activity (adjusted mean difference = 780.36, *p* <.01, *d* = 0.74) relative to CAU, whereas PLM significantly improved physical health-related QoL (adjusted mean difference = 0.27, *p* <.05, *d* = 0.66), psychological health-related QoL (adjusted mean difference = 0.45, *p* <.05, *d* = 0.93), and interpersonal relationships (adjusted mean difference = 3.69, *p* <.01, *d* = 0.88) compared to CAU at Week 19. Both LM/S and PLM did not differ significantly relative to CAU on functional impairment, social relationship-related QoL, overall QoL, general health, health responsibility, physical activity as assessed by the HPLP-II, or IPAQ-assessed total, moderate, and walking activity levels (*p*s > 0.05) at Weeks 7 and 19.

### Clinical significance

From Week 0 to Week 7, a minimum of 5-point reduction in the PHQ-9 was observed in 3 out of 14 participants (21.43%) from the LM/S group, 6 out of 17 participants (35.29%) from the PLM group, and 4 out of 16 participants (25%) from the CAU group. The Fisher’s exact test indicated no significant group difference in the proportion of participants who had achieved a clinically significant reduction of depressive symptoms (*p* =.71). Regarding participants who achieved a PHQ-9 total score of < 10 at Week 7, 8 out of 14 individuals in the LM/S group achieved this threshold, in comparison to 13 out of 17 individuals in the PLM group and 6 out of 16 individuals in the CAU group. Likewise, the Fisher’s exact test indicated no significant difference in the proportion of participants who had achieved a PHQ-9 total score of < 10 between the 3 groups (*p* =.09).

### Intervention credibility and expectancy

The RLMM analyses revealed significant differences in intervention credibility (*p* <.05) and expectancy (*p* <.01) between LM/S and PLM (Table 3). Specifically, participants in the PLM reported significantly higher credibility (*d* = 0.47) and expectancy (*d* = 0.32) for the multicomponent LM intervention compared to those in the LM/S group, with small effect sizes.

### Acceptability of smartphone-delivered EMA

During the 6 intervention weeks (i.e., 42 days), 3 out of 15 participants (i.e., 20%) who had started the intervention did not provide any EMA responses. In addition, 6 instances of participants not receiving EMA prompts were reported, with 4 occurred in Week 2 and the remaining 2 took place in Week 5 and Week 6, respectively. In sum, 3,753 EMA responses out of the 13,860 EMA prompts sent (27.1%) were collected from 15 participants. Among these participants, 3 were classified as ‘completers’ for responding to ≥ 30% of the total EMA measures, while the remaining 12 were deemed ‘non-completers’. A comparison of baseline clinical, lifestyle, and demographic characteristics between ‘completers’ and ‘non-completers’ is shown in Supplementary Table 2. No baseline characteristics displayed significant differences between the 2 groups. A Friedman test was conducted to investigate potential ‘fatigue effects’ on a weekly basis. The findings indicated no significant difference in the number of EMA responses completed from Week 1 through Week 6 [*x*^2^(5) = 9.77, *p* =.08)] (Supplementary Fig. 1).

### Post-hoc power analysis

Post-hoc power analyses were conducted for the primary outcome (PHQ-9) at Week 7 across the three groups (LM/S, PLM, and CAU). Based on the observed effect size between LM/S and PLM (*d* = 0.54), LM/S and CAU (*d* = 0.24), and PLM and CAU (*d* = 0.78), the achieved powers were 0.53, 0.28, and 0.71, respectively.

**Table 2:** Effects of group LM intervention at the immediate post-intervention (Week 7) and 3-month follow-up (Week 19) assessments (based on ITT principle).

Measure	LM/S vs. PLM at Week 7 Δ estimated mean (SE)	*p*-value (*d*)	LM/S vs. CAU at Week 7 Δ estimated mean (SE)	*p*-value (*d*)	PLM vs. CAU at Week 7 Δ estimated mean (SE)	*p*-value (*d*)
PHQ-9						
Week 7	2.75 (1.80)	.28 (0.54)	−1.21 (1.93)	.80 (0.24)	−3.96 (1.71)	.05 (0.78)
Week19	−1.78 (1.78)	.58 (0.35)	**−5.80 (1.93)**	< .01** (1.14)	**−4.02 (1.71)**	< .05* (0.79)
GAD-7						
Week 7	2.50 (1.82)	.36 (0.52)	−0.56 (1.96)	.96 (0.12)	−3.05 (1.75)	.19 (0.64)
Week19	−0.40 (1.83)	.97 (0.08)	**−5.22 (1.99)**	< .05* (1.09)	**−4.82 (1.76)**	< .05* (1.01)
ISI						
Week 7	3.53 (1.73)	.10 (0.74)	−1.87 (1.85)	0.57 (0.39)	**−5.40 (1.66)**	< .01** (1.13)
Week19	−0.68 (1.73)	.92 (0.14)	**−6.90 (1.88)**	< .001*** (1.44)	**−6.21 (1.66)**	< .001*** (1.30)
SDS						
Week 7	2.03 (2.63)	.72 (0.26)	−1.63 (2.81)	.83 (0.21)	−3.65 (2.54)	.32 (0.48)
Week19	0.45 (2.64)	.98 (0.06)	−3.51 (2.87)	.44 (0.46)	−3.96 (2.54)	.26 (0.52)
QoL - physical health						
Week 7	−0.24 (0.11)	.07 (0.58)	0.08 (0.11)	.79 (0.18)	**0.32 (0.11)**	< .01** (0.76)
Week19	−0.10 (0.11)	.61 (0.25)	0.17 (0.12)	.32 (0.41)	**0.27 (0.11)**	< .05* (0.66)
QoL - psychological health						
Week 7	−0.19 (0.18)	.53 (0.39)	0.01 (0.19)	1.00 (0.01)	0.20 (0.17)	.48 (0.41)
Week19	−0.09 (0.18)	.87 (0.19)	0.36 (0.19)	.15 (0.74)	**0.45 (0.17)**	< .05* (0.93)
QoL - social relationship						
Week 7	−0.33 (0.29)	.49 (0.45)	0.02 (0.31)	1.00 (0.03)	0.35 (0.28)	.42 (0.48)
Week19	−0.14 (0.29)	.88 (0.20)	0.47 (0.31)	.29 (0.65)	0.61 (0.28)	.07 (0.85)
QoL - environmental health						
Week 7	0.14 (0.19)	.75 (0.23)	**0.55 (0.21)**	< .05* (0.92)	0.41 (0.19)	.07 (0.69)
Week19	0.23 (0.19)	.45 (0.39)	**0.74 (0.21)**	< .01** (1.23)	**0.50 (0.19)**	< .05* (0.84)
QoL – overall quality of life						
Week 7	−0.30 (0.26)	.47 (0.40)	0.11 (0.28)	.92 (0.14)	0.42 (0.25)	.23 (0.54)
Week19	0.22 (0.26)	.68 (0.28)	0.65 (0.28)	.06 (0.84)	0.43 (0.25)	.21 (0.56)
QoL - general health						
Week 7	−0.25 (0.35)	.75 (0.36)	−0.07 (0.38)	.98 (0.10)	0.19 (0.34)	.85 (0.26)
Week19	0.70 (0.35)	.12 (0.98)	0.64 (0.38)	.21 (0.90)	−0.05 (0.34)	.99 (0.08)
HPLP-II overall lifestyle						
Week 7	−10.41 (6.39)	.23 (0.56)	6.36 (6.90)	.63 (0.34)	**16.77 (6.13)**	< .05* (0.90)
Week19	0.91 (6.40)	.99 (0.05)	**21.16 (6.98)**	< .01** (1.14)	**20.25 (6.13)**	< .01** (1.09)
HPLP-II Health responsibility						
Week 7	−0.59 (1.71)	.94 (0.14)	1.59 (1.85)	.67 (0.36)	2.18 (1.65)	.38 (0.50)
Week19	0.29 (1.72)	.98 (0.07)	2.40 (1.87)	.40 (0.55)	2.11 (1.65)	.41 (0.48)
HPLP-II Physical activity						
Week 7	−1.11 (1.53)	.75 (0.26)	0.98 (1.65)	.82 (0.23)	2.09 (1.47)	.33 (0.48)
Week19	0.53 (1.53)	.94 (0.12)	3.15 (1.67)	.14 (0.73)	2.62 (1.47)	.17 (0.60)
HPLP-II Nutrition						
Week 7	−3.49 (1.83)	.14 (0.72)	0.85 (1.98)	.90 (0.18)	**4.34 (1.75)**	< .05* (0.90)
Week19	1.23 (1.83)	.78 (0.26)	**4.81 (2.00)**	< .05* (1.00)	3.58 (1.75)	.10 (0.74)
HPLP-II Spiritual growth						
Week 7	−0.93 (1.64)	.84 (0.20)	1.20 (1.78)	.78 (0.26)	2.12 (1.57)	.36 (0.46)
Week19	0.81 (1.64)	.87 (0.17)	**4.44 (1.79)**	< .05* (0.95)	3.62 (1.57)	.05 (0.78)
HPLP-II Interpersonal relationship						
Week 7	−1.39 (1.31)	.54 (0.33)	0.18 (1.41)	.99 (0.04)	1.56 (1.26)	.43 (0.37)
Week19	−0.78 (1.31)	.82 (0.19)	2.91 (1.43)	.10 (0.69)	**3.69 (1.26)**	< .01** (0.88)
HPLP-II Stress management						
Week 7	−2.18 (1.20)	.17 (0.64)	0.89 (1.29)	.77 (0.26)	**3.07 (1.16)**	< .05* (0.89)
Week19	0.76 (1.21)	.81 (0.22)	**3.62 (1.31)**	< .05* (1.05)	**2.86 (1.16)**	< .05* (0.83)
IPAQ Total activity						
Week 7	−519.22 (563.98)	.63 (0.26)	−14.62 (601.39)	1.00 (0.01)	504.60 (545.32)	.62 (0.25)
Week19	66.73 (586.45)	.99 (0.03)	394.88 (615.13)	.80 (0.20)	328.15 (563.70)	.83 (0.16)
IPAQ Vigorous activity						
Week 7	138.20 (235.01)	.83 (0.13)	390.48 (244.78)	.25 (0.37)	252.27 (229.56)	.51 (0.24)
Week19	**745.03 (248.75)**	< .01** (0.71)	**780.36 (253.40)**	< .01** (0.74)	35.33 (240.10)	.99 (0.03)
IPAQ Moderate activity						
Week 7	−52.95 (111.57)	.88 (0.09)	−17.91 (118.44)	.99 (0.03)	35.03 (108.09)	.94 (0.06)
Week19	50.90 (116.57)	.90 (0.09)	138.37 (121.51)	.49 (0.24)	87.48 (112.14)	.72 (0.15)
IPAQ Walking						
Week 7	−517.60 (277.74)	.15 (0.52)	−309.97 (292.25)	.54 (0.31)	207.64 (270.09)	.72 (0.21)
Week19	−290.57 (292.41)	.58 (0.29)	−60.29 (301.35)	.98 (0.06)	230.28 (281.69)	.69 (0.23)

CAU, care-as-usual: CEQ, Credibility-Expectancy Questionnaire; LM/S, EMA-supported group multicomponent LM intervention: GAD-7, Generalized Anxiety Disorder-7 assessment; HPLP-II, Health-Promoting Lifestyle Profile; IPAQ, International Physical Activities Questionnaire – Chinese version; ISI, Insomnia Severity Index; ITT, intention to treat; PHQ-9, Patient Health Questionnaire; PLM, pure multicomponent LM intervention; SDS, Sheehan Disability Scale; SE, standard error; WHOQOL-brief, World Health Organization Quality of Life Instruments.

* *p* <.05.

** *p* <.01.

*** *p* <.001.

**Table 3:** Intervention credibility and expectancy.

Measure	LM/S (*n* = 18) Predicted mean (SE)	PLM (*n* = 20) Predicted mean (SE)	*p*-value (*d*)
CEQ-credibility			
Week 0	18.94 (1.12)	16.76 (1.07)	
Week 7	17.83 (1.21)	20.33 (1.24)	< .05* (0.47)
CEQ-expectancy			
Week 0	16.68 (1.05)	13.50 (1.01)	
Week 7	14.09 (1.13)	15.68 (1.15)	< .01** (0.32)

CEQ, Credibility-Expectancy Questionnaire; LM/S, EMA-supported group multicomponent LM intervention; PLM, pure multicomponent LM intervention; SE, standard error.

* *p* <.05.

** *p* <.01.

## Discussion

While the LM approach has garnered increasing attention for the management of depression, a major implementation challenge lies in motivating individuals with depression, who often exhibit low motivation, to adhere to the multicomponent LM intervention. This pilot RCT represents the first investigation of using smartphone-delivered EMA as a self-monitoring tool to enhance adherence to a group-based, multicomponent LM intervention aimed at ameliorating depressive symptoms. However, the preliminary findings indicated that LM/S did not differ significantly from PLM in improving depressive symptoms at either Week 7 or Week 19. Similarly, LM/S showed no significant effect on depressive symptoms compared with CAU at Week 7, whereas PLM demonstrated a marginally significant reduction compared to CAU. No significant differences were observed between groups in the proportion of participants achieving a clinically significant reduction in depressive symptoms at Week 7. In contrast, both intervention groups exhibited a trend of significantly greater reductions in depressive symptoms than CAU at Week 19, with moderate to large effect sizes. Regarding secondary outcomes, the LM/S only showed a trend of significantly large improvements in environmental health-related QoL than the CAU at Week 7, whereas PLM demonstrated moderate to large improvements in insomnia symptoms, physical health-related QoL, overall lifestyle, nutrition, and stress management. At Week 19, both intervention groups showed significant improvements in anxiety symptoms, insomnia symptoms, environmental health-related QoL, overall lifestyle, and stress management relative to CAU. Moreover, the LM/S showed additional improvements in nutrition, spiritual growth, and IPAQ measured vigorous activity level relative to CAU, whereas PLM showed gains in physical health- and psychological-related QoL and interpersonal relationships. The only significant difference between the 2 intervention groups was that LM/S showed higher IPAQ-measured vigorous activity at Week 19. Regarding intervention acceptability, PLM participants rated significantly higher than LM/S on both intervention credibility and intervention expectancy, indicating greater confidence in and anticipated benefit from the pure multicomponent LM intervention. Within LM/S, engagement with EMA was modest, with a 27.1% response rate, and there was no evidence of baseline differences between EMA completers and non-completers or of weekly fatigue effects.

The preliminary findings suggest that both LM/S and PLM are more efficacious than CAU in reducing depressive and anxiety symptoms at Week 19. These findings algin with existing meta-analyses of multicomponent LM interventions, as well as our prior RCT on group-based multicomponent LM intervention for individuals with probable depressive disorder, which collectively report small to large effect sizes compared to inactive control groups at short-term follow-up (*d* = 0.25 to 0.78)^11,12^. However, the lack of significant effects on depressive and anxiety symptoms in both intervention groups at immediate post-intervention (i.e., Week 7) differs from these earlier studies. One plausible explanation for these discrepancies is that the present study was conducted amidst the fifth wave of coronavirus disease (COVID-19) in Hong Kong (i.e., 31 December 2021 to 29 January 2023). The ongoing pandemic-induced pressures, such as heightened uncertainty, social isolation, and constrained opportunities for physical activity due to lockdown (Luchetti et al., 2020), may have masked early intervention gains in the LM/S and PLM groups. Consequently, participants in the current trial may have required additional time for the cumulative benefits of multicomponent LM intervention to overcome these external stressors, as evidenced by the significant improvements observed at Week 19. Another explanation is that the pilot nature of this study resulted in insufficient statistical power to detect small between-group differences, thereby elevating the risk of Type II errors. As such, the existing findings should be interpreted with caution, with full-scale trials needed to confirm whether the observed findings can be replicated in larger samples.

Regardless of these situational factors and methodological limitations, the observed preliminary findings indicated that PLM has resulted in broader improvements in insomnia symptoms, physical health-related QoL, overall lifestyle, nutrition, and stress management compared to CAU at Week 7, whereas the LM/S only demonstrated significant improvement in environment-related QOL during the same period. One possibility is that the initial learning phase associated with the smartphone-delivered EMA application, or the process of self-monitoring lifestyle behaviors and mood, may have temporarily influenced participants’ full engagement in lifestyle modifications. As participants in the LM/S group became more familiar with the EMA and self-monitoring process over time, they may have begun to experience benefits from the multicomponent LM intervention comparable to those observed in the PLM group at Week 19. Another potential explanation is that the integration of smartphone-delivered EMA might have inadvertently introduced distractions or impediments to the lifestyle modification process. However, this seems less likely given the absence of reported burdensome experiences related to the EMA design in this study and the broader meta-analytic evidence supporting its acceptability as a self-monitoring tool for promoting health behaviors^59–62^. While these possibilities remain speculative, the pilot nature of this study underscores the need for caution in interpreting these findings. Future research with longer follow-up periods will be essential to better understand the potential mechanisms underlying these treatment response patterns.

Moreover, the findings showed comparable study attrition (15%–22.22%) between groups, aligning with recent meta-analyses examining the effect of multicomponent LM interventions for improving depressive symptoms, which reported attrition rates ranging from 20.6% to 22%^11,18^. Additionally, both LM/S and PLM demonstrated an attendance rate of over 80%, which is higher than the 66% attendance rate reported for prior multicomponent LM interventions specifically targeting depression^18^. Although the proportion of participants who demonstrated full adherence to intervention sessions was higher in the LM/S (66.67%) than in the PLM (38.89%), the difference between the 2 intervention groups was not statistically significant. In comparison to existing multicomponent LM interventions for depression (53%), the LM/S group exhibited a greater proportion of participants who demonstrated full adherence to the intervention, whereas the PLM group showed a smaller proportion of participants with full adherence to the intervention (38.89%). These findings stand in contrast to our previously conducted group-based multicomponent LM interventions, in which a mere 12.5% of participants attended all treatment sessions^12^.

Regarding the response rate for smartphone-delivered EMA, the LM/S group exhibited a relatively lower response rate of 27.1%. This is in contrast to previous meta-analyses that investigated EMA interventions for promoting health behaviors in both general and depressed populations, which reported a pooled response rate of approximately 80% (range = 30.7% to 87%)^59–62^. The low EMA response rate, combined with the significantly lower credibility (*d* = 0.47) and expectancy (*d* = 0.32) of the LM/S, could be reflective of several issues related to our EMA implementation and design. First, unforeseeable technical problems are one of the main reason to deter participants from consistently engaging with EMA, leading to a reduced response rate^59^. In our study, although there were no reports of crashing of the EMA smartphone application, six instances of participants not receiving EMA prompts were reported. It is essential for future trials to ensure a smooth EMA delivery, such that the overall connectedness and satisfaction of the EMA experience could be improved, ultimately leading to better engagement and more accurate data collection. Second, the absence of incentives for EMA responses may have also contributed to the low response rate in the LM/S^63^. This is particularly notable given that our EMA period lasted 42 days, which is comparatively longer than existing EMA trials designed to promote health behaviors in general and depressed populations (range = 3–30 days, median = 7–14 days)^59,61–63^. However, this mirrors the use of EMA in real-world clinical practice to promote health behaviors, where incentives are unlikely to be provided. Third, the interval-contingent design of the smartphone-delivered EMA may be overly intensive and burdensome for quantifying infrequent events, such as exercising, eating occasion, and practicing stress management techniques^64^, even though no reports of burden regarding the smartphone-delivered EMA were received. Future trials could consider adopting an event-contingent design to better capture these less frequent occurrences and reduce participant burden^61^. Additionally, subsequent studies could investigate the potential differential effects of employing various EMA sampling protocols (e.g., interval-contingent, signal-contingent, and event-contingent designs) as a self-monitoring tool for lifestyle and mood in supporting multicomponent LM interventions for depression. Furthermore, integrating wearable devices into such protocols could provide continuous, objective, and real-time data on lifestyle factors (e.g., physical activity, sleep) and physiological states, complementing self-reported EMA data. Besides, future research could focus on developing just-in-time adaptive interventions (JITAIs) to support lifestyle modifications for depressive symptoms as well as adopting micro-randomized trials to optimize JITAIs.

This was the first pilot RCT to explore the application of smartphone-delivered EMA as a self-monitoring tool to augment a group-based, multicomponent LM intervention targeting the improvement of depressive symptoms. The findings of this study provide a crucial foundation for refining future intervention designs and estimating sample size in larger scale RCTs. However, several limitations should be acknowledged. First, as mentioned, it is important to note that the present study was conducted amidst the fifth wave of COVID-19 in Hong Kong. Consequently, the generalizability of our findings to a post-COVID-19 context may be limited. Second, this trial served as a pilot study to evaluate the EMA-supported multicomponent LM intervention. As such, the robustness of our findings was limited by the small sample size, and the results should be interpreted with caution. Third, we did not perform moderator analyses to identify sociodemographic and participant characteristics that might be associated with differential intervention effects given the small sample size. Fourth, although a community sample recruitment strategy was used, the sample was predominantly female (85.7%) and a significant majority (80.4%) did not have children. As such, the generalizability of our findings may be limited. Fifth, the trial design made blinding of participants and research personnel not possible, which could potentially introduce performance and detection bias. Finally, depressive symptoms in this pilot RCT were assessed solely with the PHQ-9. For a future definitive trial, we may augment the PHQ‑9 with a more comprehensive measure, such as the Beck Depression Inventory–II^65^, to capture a broader range of symptom domains and enhance the evaluation of intervention effects.

In summary, the preliminary findings suggest that a group-based, multicomponent LM intervention may be efficacious for improving depressive symptoms, and that smartphone-delivered EMA has the potential to enhance full adherence to multicomponent LM intervention. However, it is recognized that the delivery and format of this technology need to be fine-tuned. Specifically, future studies should enhance the EMA prompt delivery and adopt an event-contingent design to boost EMA compliance. A future adequately powered trial is warranted to evaluate the benefits of an EMA-supported, multicomponent LM intervention for depression.

## Supplementary Information

Below is the link to the electronic supplementary material.

Supplementary Material 1

## Data Availability

The data that support the findings of this study are available from the corresponding author, FYH, upon reasonable request.
