# Supplementary material for: Enhancing group lifestyle intervention for depression with ecological momentary assessment: a pilot randomized controlled trial
Source: Sci Rep. 2025 Oct 29;15:37818. doi: 10.1038/s41598-025-21688-x (PMC12572295; doi:10.1038/s41598-025-21688-x)

Supplementary Table 1 Overview of the 6-week Group-based Lifestyle Medicine Intervention.

| Program Structure | | | |
| --- | --- | --- | --- |
| Goal Setting | Behavior Change and Motivation | Session 1 | *Program overview*  • Basic facts about depression and lifestyle intervention  • The relationship between lifestyle factors and depression  *Diet and nutrition (I)*  • Diet and mental health - what is the link?  • Understanding and using nutrition facts labels (demonstration and practice)  • Action steps for monitoring unhealthy diet |
|  |  | Session 2 | *Physical activity (I)*  • Physical and psychological benefits  • Physical activity recommendations  • Low-intensity home-based exercise (demonstration and practice)  *Stress management (I)*  • Psychoeducation on stress  • Diaphragmatic breathing |
|  |  | Session 3 | *Sleep management (I)*  • Sleep hygiene  • Establishing regular sleep-wake times  • Learning a wind-down routine  *Stress management (II)*  • Types of worries  • Worry time (demonstration and practice) |
|  |  | Session 4 | *Diet and nutrition (II)*  • Integrating healthy diet into daily lives  • Action steps for a healthy diet  • Helpful meal preparation tips  *Stress management (III)*  • Progressive muscle relaxation |
|  |  | Session 5 | *Physical activity (II)*  • Moderate-intensity physical activity (demonstration and practice)  *Sleep management (II)*  • Learning a wake-up routine  • Improving daytime functioning |
|  |  | Session 6 | *Socializing*  • Importance of social networking  • Regularizing social rhythm  • Tips for socializing when depressed  *Stress management (IV)*  • Scientific values of positive psychology  • Practical skills in cultivating gratitude  *Program review* |

Supplementary Table 2 Baseline characteristics between ‘completer’ and ‘non-completer’.

| Variable | Completer (*n* = 3) | Non-completer (*n* = 12) | Total (*n* = 15) | *p*-value |
| --- | --- | --- | --- | --- |
| Age, years | 42 (10) | 40 (15) | 40 (15.5) | .83 |
| Female, n (%) | 2 (66.67) | 10 (83.33) | 12 (80) | .52 |
| Marital status, n (%) |  |  |  | .53 |
| Not married | 1 (33.33) | 8 (66.67) | 9 (60) |  |
| Married | 2 (66.67) | 4 (33.33) | 6 (40) |  |
| Divorced/widowed | 0 (0) | 0 (0) | 0 (0) |  |
| Educational level, n (%) |  |  |  | 1 |
| Secondary or below | 2 (66.67) | 6 (50) | 8 (53.33) |  |
| Associate degree, diploma, or vocational training | 0 (0) | 3 (25) | 3 (20) |  |
| Bachelor’s degree | 1 (33.33) | 3 (25) | 4 (26.67) |  |
| Master’s degree or above | 0 (0) | 0 (0) | 0 (0) |  |
| Number of children, n (%) |  |  |  | .23 |
| 0 | 2 (66.67) | 10 (83.33) | 12 (80) |  |
| 1 | 0 (0) | 2 (16.67) | 2 (13.33) |  |
| ≥2 | 1 (33.33) | 0 (0) | 1 (6.67) |  |
| Employment status, n (%) |  |  |  | 1 |
| Full-time work/student | 2 (66.67) | 8 (66.67) | 10 (66.67) |  |
| Part-time work | 0 (0) | 1 (8.33) | 1 (6.67) |  |
| Unemployed | 0 (0) | 0 (0) | 0 (0) |  |
| Retired or carer | 1 (33.33) | 3 (25) | 4 (26.67) |  |
| Monthly income, n (%) |  |  |  | 1 |
| ≤HKD$ 5,000 | 1 (33.33) | 4 (33.33) | 5 (33.33) |  |
| HKD$ 5,001–10,000 | 0 (0) | 2 (16.67) | 2 (13.33) |  |
| HKD$ 10,001–20,000 | 1 (33.33) | 1 (8.33) | 2 (13.33) |  |
| HKD$ 20,001–30,000 | 0 (0) | 2 (16.67) | 2 (13.33) |  |
| HKD$ 30,001–50,000 | 1 (33.33) | 2 (16.67) | 3 (20) |  |
| HKD$ 50,001–70,000 | 0 (0) | 1 (8.33) | 1 (6.67) |  |
| HKD$ 70,001–90,000 | 0 (0) | 0 (0) | 0 (0) |  |
| >HKD$ 90,000 | 0 (0) | 0 (0) | 0 (0) |  |
| Current antidepressant medication, n (%) | 0 (0) | 2 (16.67) | 2 (13.33) | 1 |
| PHQ-9 | 10(5.5) | 9 (5.75) | 9 (7.5) | 1 |
| GAD7 | 15 (6) | 11.5 (9) | 13 (9) | .88 |
| ISI | 15(3.5) | 16(6.25) | 16 (6) | .77 |
| SDS | 11 (6.5) | 8.5 (14.75) | 9 (13.5) | .72 |
| WHOQOL-brief |  |  |  |  |
| Physical health | 2.86 (0.64) | 2.93 (0.36) | 2.86 (0.36) | .51 |
| Psychological health | 2.33 (0.33) | 2.83 (1.12) | 2.83 (0.92) | .42 |
| Social relationship | 3 (1) | 3 (0.75) | 3 (0.83) | .65 |
| Environmental health | 2.28 (0.69) | 3.25 (0.66) | 3.12 (1) | .17 |
| Quality of life | 3 (0.5) | 3 (1.25) | 3 (1.5) | .40 |
| General health | 2 (0) | 3 (1) | 2 (1) | .17 |
| HPLP-II |  |  |  |  |
| Total score | 96 (5) | 108.5 (19.25) | 107 (18.5) | .10 |
| Health responsibility | 15 (3.5) | 18.5 (5) | 17 (4.5) | .15 |
| Physical activity | 11 (0.5) | 17 (5.75) | 16 (7) | .11 |
| Nutrition | 21 (2. 5) | 20 (5) | 21 (4.5) | .77 |
| Spiritual growth | 16(2) | 18.5 (4.25) | 18 (4.5) | .47 |
| Interpersonal relationship | 18 (2.5) | 20 (2.5) | 20 (2.5) | .42 |
| Stress management | 15 (2) | 15.5 (4.25) | 15 (4) | .88 |
| IPAQ |  |  |  |  |
| Total activity | 198 (513) | 3492 (3774) | 2325 (4200) | .10 |
| Vigorous activity | 0 (480) | 720 (640) | 720 (960) | .34 |
| Moderate activity | 0 (0) | 240 (960) | 80 (600) | .09 |
| Walking | 198 (33) | 1980 (1782) | 1485 (2227.5) | .06 |
| CEQ |  |  |  |  |
| Credibility | 16 (3.5) | 19.5 (5.75) | 19 (6.5) | .61 |
| Expectancy | 15 (6.5) | 17.5 (5.5) | 17 (5) | .34 |

CAU, care-as-usual: CEQ, Credibility-Expectancy Questionnaire; ELM, EMA-supported group multicomponent LM intervention: GAD-7, Generalized Anxiety Disorder-7 assessment; HPLP-II, Health-Promoting Lifestyle Profile; IPAQ, International Physical Activities Questionnaire – Chinese version; ISI, Insomnia Severity Index; PHQ-9, Patient Health Questionnaire; PLM, pure multicomponent LM intervention; SDS, Sheehan Disability Scale; WHOQOL-brief, World Health Organization Quality of Life Instruments.

*Note*. Data are presented as median and interquartile range or frequency and percentage.

Supplementary Figure 1 Ecological momentary assessment response rates throughout the 6 intervention weeks (*n* = 15).


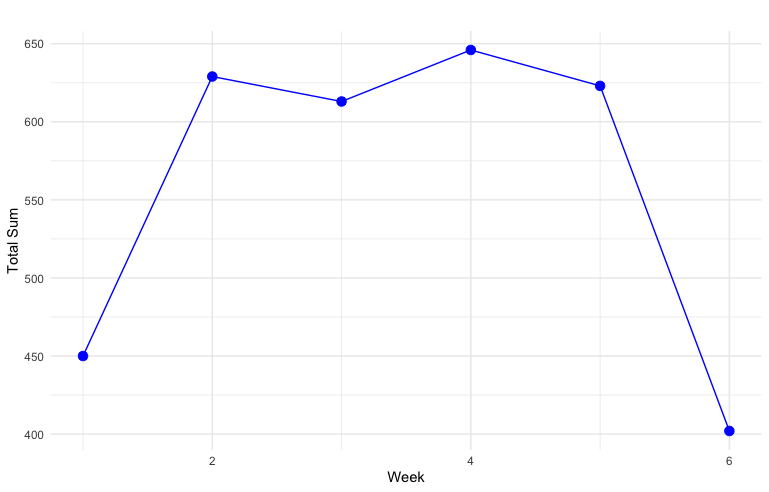

Supplement: Supplementary file 1 — Supplementary Material 1 [file 41598_2025_21688_MOESM1_ESM.docx]
